# Effect of Endomorphins on HUVECs Treated by ox-LDL and Its Related Mechanisms

**DOI:** 10.1155/2016/9741483

**Published:** 2016-08-04

**Authors:** Juan Zhao, Qi Zhang, Jing Liu, Liming Tian, Wenhui Huang, Jinxing Quan, Jinyang Wang, Yanjia Xu, Yunfang Wang, Ruilan Niu

**Affiliations:** ^1^Department of Endocrinology, Gansu Provincial Hospital, 204 West Donggang Road, Lanzhou, Gansu 730000, China; ^2^Department of Nephrology, Gansu Provincial Hospital, 204 West Donggang Road, Lanzhou, Gansu 730000, China

## Abstract

We found in the present study that treatment with ox-LDL decreased the cell viability and the content of nitric oxide (NO) and the activity of nitric oxide synthase (NOS) as well as eNOS mRNA expression, while increasing the mRNA expression and content of endothelin-1 (ET-1) in human umbilical vein endothelial cells (HUVECs). However, endomorphins EM1/EM2 increased the cell viability and the content of NO and the activity of NOS as well as eNOS mRNA expression, while decreasing the mRNA expression and content of ET-1 compared with ox-LDL alone. Meanwhile, the expressions of JNK and p-JNK were enhanced by ox-LDL while being suppressed by EM1/EM2. The results suggested that EM1 and EM2 can correct the endothelial cell dysfunction induced by ox-LDL and the protective effect may be achieved by affecting the JNK pathway.

## 1. Introduction

Diabetes mellitus (DM) is a common endocrine disease, and its various acute or chronic complications greatly threaten human's life [1]. Vascular lesion is the most important complication of DM and plays a crucial role in other complications. The pathogenesis of the vascular lesions is related to the dysfunction of vascular endothelial cell, platelet activation, abnormality of coagulation, and fibrinolysis, of which the dysfunction of vascular endothelial cell is the key symptom of vascular lesions at its early stage and initial factor of the development of arteriosclerosis [2]. For those who suffered from DM, long-term hyperglycemia and dyslipidemia are important environmental factors for vascular endothelial cell, both of which could cause the dysfunction of vascular endothelial diastolic factor and thus further harm the function of endothelial cell and can also contribute to the heart and blood vessel complications of diabetes [3, 4]. Among endogenous opioid peptides, endomorphins EM1 and EM2 are two potent and highly selective *μ*-opioid receptor agonists which were found in mammal nervous system and possess general biological and pharmacological activities. By coupling to G-protein, they can adjust pain, cardiovascular function, and immune function [5]. The experiment showed that EM1/EM2 had apparent vasodilating effects on vascular bed of rat's hind limb [6]. Further research indicated that vasodilating effects resulted from nitric oxide (NO) released by vascular endothelial cell [7]. Besides, NO plays an important role in maintaining normal vascular endothelial function; therefore, it was speculated that EM1/EM2 can exert influence on diabetic endothelial cells.

By observing the impacts of EM1/EM2 with different drug concentration on NO, nitric oxide synthase (NOS), and endothelin-1 (ET-1), the present study was conducted to investigate the effects of EM1/EM2 on the mechanism of vascular endothelial cells under ox-LDL.

## 2. Materials and Methods

### 2.1. Materials

EM1/EM2 (purity > 99%) and SP600125 were purchased from Shanghai Hanhong Chemical Co., Ltd. (Shanghai, China); DMEM and fetal bovine serum (FBS) were purchased from Gibco-BRL Company (Gibco, NY, USA); NO assay kit, NOS detection kit, and ET-1 enzyme-linked immunosorbent assay kit were purchased from Nanjing Jiancheng Bioengineering Institute (China). RT-PCR kit was purchased from TaKaRa Bio Inc. (Dalian, China). Rabbit anti-phospho-JNK antibody and FITC-conjugated secondary antibodies were purchased from Bioworld Technology (Inc., USA).

### 2.2. Cell Culture and Treatment

Human umbilical vein endothelial cells (HUVECs) were isolated from fresh human umbilical cords according to the method of Jaffe et al. [8] with the approval of Ethics Committee of Lanzhou Medical College. Cells were cultured in low-glucose DMEM (5.5 mM glucose) supplemented with 10% fetal calf serum, benzylpenicillin (100 U/mL), and streptomycin (100 *μ*g/mL). Then they were incubated in humidified atmosphere with 5% CO_2_ and 95% air at 37°C. Cells were identified as HUVECs in the presence of factor VIII antigen and their typical cobblestone morphology. Passages 3–5 of HUVECs were used in the course of the experiment. HUVECs were cultured and divided into seven groups: Group A: normal culture group; Group B: cells cultured in ox-LDL (80/100 mg/L) group; Group C: cells treated with EM1/EM2 (10 nmol/L); Group D: cells treated with EM1/EM2 (100 nmol/L); Group E: cells treated with EM1/EM2 (1000 nmol/L); Group F: cells treated with EM1/EM2 (10000 nmol/L); Group G: cells treated with 10.0 *μ*M SP600125 + EM1/EM2 (10000 nmol/L). G group were pretreated by SP600125 for 1 hour and then cultivated by high concentration of EMs for 48 hours continuously; from C group to F group, they were cultivated by different concentrations of EM1/EM2 and ox-LDL. Our preliminary research determined the concentration of the EM [9].

### 2.3. MTT Assay for Cell Viability

MTT assay was used to determine cell viability. HUVECs were incubated into 12-well plates at a density of 5 × 10^4^ cells/well, when adhering to the surface and forming a monolayer; JNK inhibitor SP600125 was added to preculture for 1 h; then EM1/EM2 was added for 2 h and then incubated for 48 h under ox-LDL. 10 *μ*L MTT (5 mg/mL) was added to each well, and 100 *μ*L 10% SDS was added after 4 h. At last, the optical density (OD) was measured at an emission wavelength of 570 nm.

### 2.4. Evaluation of NO Production

Production of NO was evaluated with colorimetric analysis. Briefly, the cells were harvested and the media were collected; then 100 *μ*L of Griess reagent was added to the 100 *μ*L of media. After 10 mins the absorbance was measured at 570 nm and the concentration of NO was calculated.

### 2.5. Determination of NOS and eNOS

Cells were harvested and lysed by ultrasonic cell disruptor following the instruction of the commercial kit; then expression of NOS and eNOS in cells was measured according to the NOS assay's instructions.

### 2.6. Measurement of ET-1

The level of ET-1 in cells was measured using a human ET-1 enzyme-linked immunosorbent assay kit. Absorbance was measured at 450 nm with a microplate reader.

### 2.7. Real-Time RT-PCR Analysis for eNOS and ET-1 mRNA Level

Total RNA was extracted using TRIzol reagent (Invitrogen) according to the manufacture's protocol. RNA concentration was determined by using spectrophotometer (Beckman Instrument, California, USA). 2 *μ*g RNA was reverse-transcribed to cDNA with Prime Script WRT reagent kit. Real-time PCR assays were performed using The Light Cycler (Roche, New York, USA) thermocycler. Samples were denatured at 95°C for 30 s, followed by 50 PCR cycles, each cycle consisting of 95°C for 5 s and 60°C for 30 s. PCR primers were designed and synthesized from TaKaRa company. The eNOS (NM_000603.3.) primers were (forward) 5′-GCTGTCTGCATGGACCTGGA-3′ and (reverse) 5′-TCCACGATGGTGACTTTGGCTA-3′. ET-1 (NM_001955.3.) primers were (forward) 5′-GGTTCAGTTTGAACGGGAGGT-3′ and (reverse) 5′-TGGACTGGGAGTGGGTTTCT-3′. JNK (NM_002750.2) primers were 5′-CTGTGTGGAATCAAGCACCTTCA-3′ (forward) and 5′-CTGGCCAGACCGAAGTCAAGA-3′ (reverse). A housekeeping gene human *β*-actin was used to control PCR assays. *β*-actin (NM_001101.3) primer was (forward) 5′-GCAAGCAGGAGTATGACGAGT-3′ and (reverse) 5′-CTGCGCAAGTTAGGTTTTGTC-3′. The resulting data was analyzed by Rotor-Gene Real-Time Analysis Software 6.1.

### 2.8. Immunofluorescence

Immunofluorescence staining was performed as described previously [10]. Cells were fixed with 4% paraformaldehyde for 10 mins at 4°C and washed with PBS three times. The cells were permeabilized with 0.2% Triton X-100 for 5 mins at room temperature. After being blocked with 5% normal bovine serum for 30 mins, cells were incubated with p-JNK antibody (1 : 100 dilution) at 4°C overnight followed by FITC-conjugated secondary antibody (1 : 100 dilution, 1 h). Images were obtained using fluorescence microscope (IX81, Olympus, Japan).

### 2.9. Statistical Analysis

Statistical analysis was performed using SPSS 11.0. All data was expressed as mean ± SD. One-way analysis of variance (ANOVA) was adopted to express statistical analysis between two groups and least significant difference *t*-test was used to determine statistical analysis among multiple groups. Values of *P* less than 0.05 were considered to be significant.

## 3. Results

### 3.1. Effect of EM1/EM2 on Cell Viability of HUVECs Treated by ox-LDL

**Figure 1:**
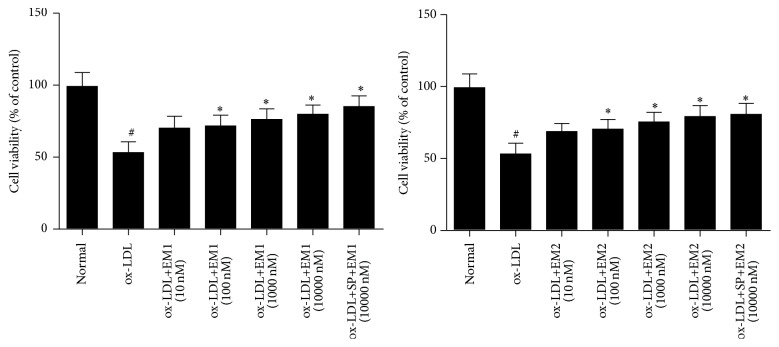
Effect of EM1/EM2 on cell viability of HUVECs treated by ox-LDL. Cell viability was determined by MTT test. All data is expressed as mean ± SD. ^*∗*^ *P* < 0.05 versus control. ^#^ *P* < 0.05 versus ox-LDL.

First, we determined HUVECs viability with MTT method. As shown in Figure 1, ox-LDL decreased the cell viability compared to normal culture group (*P* < 0.05), while cell viability can be significantly increased by EM1/EM2 compared to ox-LDL group in a concentration dependent manner. SP600125 pretreatment could increase the cell viability further (*P* < 0.05). EM1 was found to be more potent than EM2.

### 3.2. Effect of EM1/EM2 on NOS Activity of HUVECs Treated by ox-LDL

**Figure 2:**
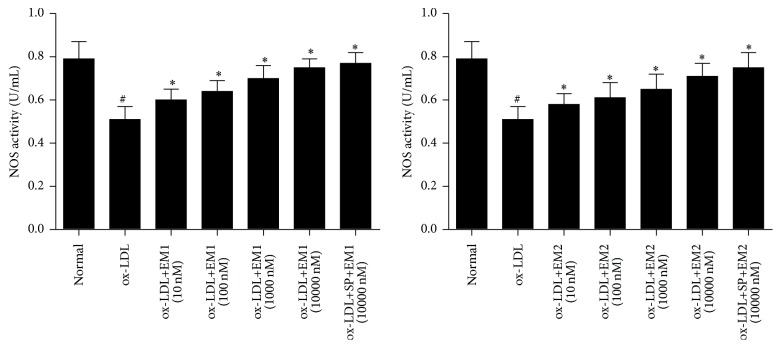
Effect of EM1/EM2 on NOS activity of HUVECs treated by ox-LDL. All data is expressed as mean ± SD. ^*∗*^ *P* < 0.05 versus control. ^#^ *P* < 0.05 versus ox-LDL.

As shown in Figure 2, the activity of NOS was decreased by ox-LDL after 48 h incubation. However, EM1 and EM2 increased the activity of NOS in a concentration dependent manner, and the activity sequence is EM1 > EM2. JNK inhibitor SP600125 increased the level of NOS further (*P* < 0.05).

### 3.3. Effect of EM1/EM2 on NO Generation of HUVECs Treated by ox-LDL

**Figure 3:**
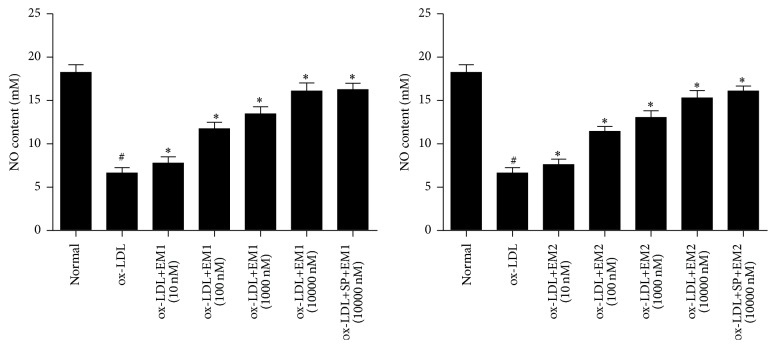
Effect of EM1/EM2 on production of NO of HUVECs treated by ox-LDL. All data is expressed as mean ± SD. ^*∗*^ *P* < 0.05 versus control. ^#^ *P* < 0.05 versus ox-LDL.

In Figure 3, the production of NO in HUVECs was decreased in the presence of ox-LDL while the production can be significantly increased by EM1/EM2 in a concentration dependent manner. JNK inhibitor SP600125 increased the level of NO further (*P* < 0.05). This was also associated with an increase in the activity of NOS.

### 3.4. Effect of EM1/EM2 on ET-I Generation of HUVECs Treated by ox-LDL

**Figure 4:**
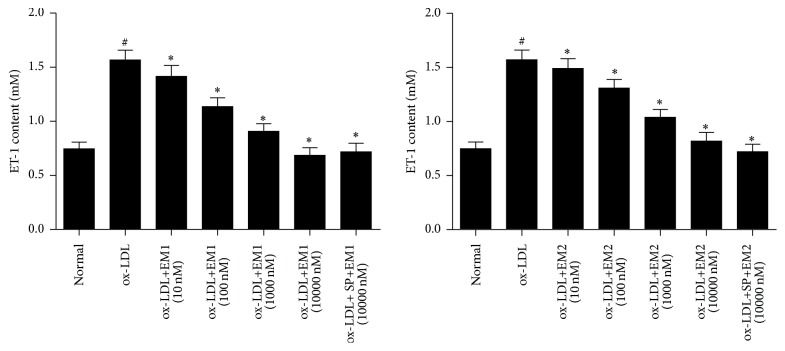
Inhibitory effect of EM1/EM2 on production of ET-1 in HUVECs treated by ox-LDL. All data is expressed as mean ± SD. ^*∗*^ *P* < 0.05 versus control. ^#^ *P* < 0.05 versus ox-LDL.

We next determined the effect of EM1/EM2 on the ET-1 production in HUVECs treated by ox-LDL. As shown in Figure 4, the level of ET-1 was increased by ox-LDL, and these enhanced expressions could be reversed in the presence of EM1/EM2 in a concentration dependent manner (*P* < 0.05). It was found that EM1 was more potent than EM2. JNK inhibitor SP600125 decreased the level of ET-I further (*P* < 0.05).

### 3.5. Effect of EM1/EM2 on mRNA Expression of eNOS in HUVECs Treated by ox-LDL

**Figure 5:**
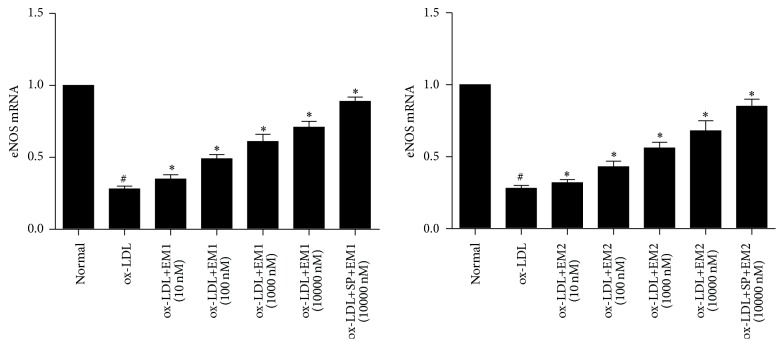
Effect of EM1/EM2 on eNOS mRNA in HUVECs treated by ox-LDL. All data is expressed as mean ± SD. ^*∗*^ *P* < 0.05 versus control. ^#^ *P* < 0.05 versus ox-LDL.

Further experiments revealed whether EM1/EM2 can increase the expression of eNOS mRNA of cells treated by ox-LDL. As shown in Figure 5, the expression of eNOS mRNA was decreased by ox-LDL, while it was increased by EM1/EM2 in a concentration dependent manner (*P* < 0.05). JNK inhibitor SP600125 increased the level of eNOS mRNA further (*P* < 0.05).

### 3.6. Effect of EM1/EM2 on mRNA Expression of ET-1 in HUVECs Treated by ox-LDL

**Figure 6:**
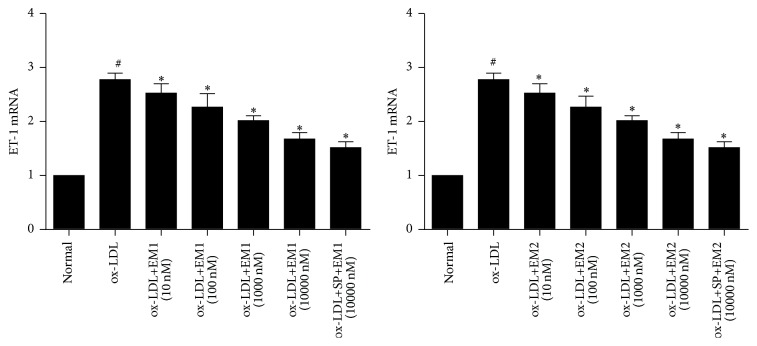
Effects of EM1 and EM2 on ET-1 mRNA expression in HUVECs. RT-PCR shows the effect of EM1 and EM2 on ET-1 mRNA expression in HUVECs in the presence or absence of ox-LDL for 48 h. Data is expressed as mean ± SD. ^*∗*^ *P* < 0.05 versus control. ^#^ *P* < 0.05 versus ox-LDL.

In Figure 6, the influence of EM1/EM2 on the ET-1 expression of HUVECs treated by ox-LDL was showed. The mRNA expression of ET-1 was increased under ox-LDL, whereas EM1/EM2 suppressed ET-1 mRNA expression (*P* < 0.05). JNK inhibitor SP600125 decreased the level of mRNA ET-1 further.

### 3.7. Effect of EM1/EM2 on Expression of JNK in HUVECs Treated by ox-LDL

**Figure 7:**
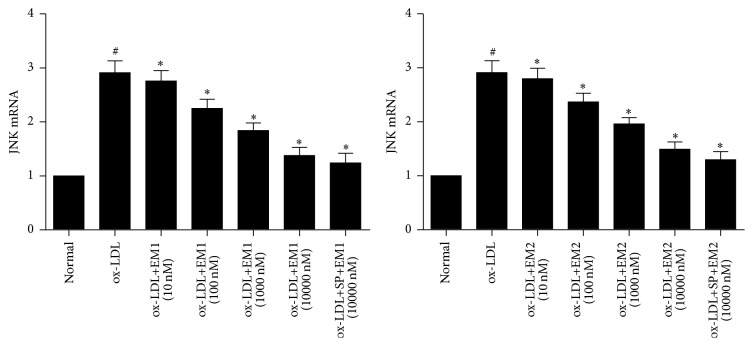
Effects of EM1 and EM2 on JNK mRNA expression in HUVECs. RT-PCR shows the effect of EM1 and EM2 on JNK mRNA expression in HUVECs induced by ox-LDL for 48 h. Data is expressed as mean ± SD. ^*∗*^ *P* < 0.05 versus control. ^#^ *P* < 0.05 versus ox-LDL.

**Figure 8:**
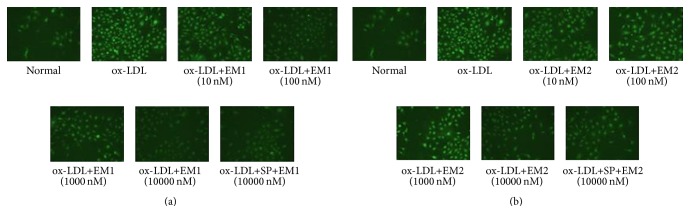
(a) Effects of EM1 on p-JNK protein in HUVECs induced by ox-LDL for 48 h by fluorescence microscope with a ×200 magnification. (b) Effects of EM2 on p-JNK protein in HUVECs induced by ox-LDL for 48 h by fluorescence microscope with a ×200 magnification.

To determine whether the effect of EM1 and EM2 on HUVECs treated by ox-LDL is related to JNK signaling pathways, we tested the expression of JNK and p-JNK by real-time quantitative RT-PCR and immunofluorescence assays, respectively. In Figure 7, real-time quantitative RT-PCR showed that stimulation of HUVECs with ox-LDL increased the expression of JNK, which was markedly prevented by EM1/EM2 in a concentration dependent manner (*P* < 0.05). In Table 1 and Figure 8, immunofluorescence assays showed that, compared with control cells, the positive granule area, average light density, and integrated light density of p-JNK in HUVECs treated by ox-LDL increased, which was blocked by EM1/EM2 efficiently. JNK inhibitor SP600125 declined the level of phosphorylation further (*P* < 0.05).

## 4. Discussion

Diabetes has the characteristics of chronic high blood glucose, lipid metabolism disorder, insulin resistance, and so forth, which increased the risk of suffering from disease of heart head blood vessel and vascular correlation significantly [11]. The vascular complications are the main reasons which lead to the death rate of diabetes and its occurrence pathologic foundation is atherosclerosis. And vascular endothelial dysfunction was considered as an important link in the process of atherosclerotic vascular disease [12]. It secretes a variety of vascular active substances which play a main role in reducing the endothelial permeability, regulating vascular tone and the endothelial permeability, inhibiting platelet aggregation and leukocyte adhesion [13]. NO and ET-1, secreted by vascular endothelial cell, are two important cell active substances and work together to maintain the normal tension of blood vessels.

NO, an endothelium-derived relaxing factor and one of the most important mediators in the regulation of endothelial cell functions, was produced by NOS via left-hand arginine (L-Arg)-N-ring phosphate guanosine (cGMP) path. It may produce potent vasodilation and inhibit the mitogenesis and proliferation of vascular smooth muscle cells. NOS mainly consists of endothelial type (eNOS), neural model (nNOS), and induction type (iNOS). NO was produced mainly by eNOS under physiological condition. When there is an induced factor, the synthesis of iNOS is induced, and a lot of NO is produced, which plays an important role in the pathological processes. NO can inhibit the activities of the cells and exert their cytotoxic effect, leading to the organization and cell damage directly [14]. Not only can decrease of NO synthetic make vasodilatation dysfunction, but its role of inhibiting the aorta smooth muscle cell proliferation and platelet aggregation is also weakened. And it can further accelerate atherosclerosis and thrombosis, which could result in a variety of pathological changes [15].

The second marker was endothelin-1 (ET-1), a potent vasoactive peptide synthesized and released by the vascular endothelium, and plays an important role in vascular remodeling and endothelial dysfunction [16]. ET-1 and NO are antagonists of blood vessel active material, and they are important in regulating the activity of blood vessels. Under the pathology conditions, excessive secretion of ET-1 results in the steady contraction of blood vessels, which reduce myocardial blood flow and increase the artery calcium ion sensitivity and instability of electrical activity. All of these potential factors can lead to the occurrence and development of atherosclerosis [17].

High glucose, AGEs, inflammatory factor, and ox-LDL are major risking factors for the development of endothelial dysfunction and progression of atherosclerosis [18]. ox-LDL, the product of LDL by oxidation modification, may exist only as a small amount in the plasma at physiological condition, but it was more likely to be oxidized from LDL in DM patients. ox-LDL damaged endothelial cell directly, leading to imbalance of ET-1/NO [19]. We have demonstrated that ox-LDL decreased the cell viability of endothelial cells and the level of NO; meanwhile, the production of NOS and the expression of eNOS were also decreased. In addition, ox-LDL increased the secretion and expression of ET-1. The results are consistent with previous research [9].

The JNK signaling pathway is a member of the mitogen activated group of protein kinases that are important signal transduction enzymes involved in many facts of cellular regulation including apoptosis. The experiment showed that JNK was not expressed in normal vascular endothelial cells, but the expression has been increased after endothelial cell was induced by ox-LDL. It was thought that the damaging effects of ox-LDL on HUVECs may be associated with JNK signaling pathways. Reports confirmed that dysfunction of islet B cell induced oxidative stress. Oxidative stress, an imbalance between the production of cell-damaging free radicals and the body's ability to neutralize them, activates JNK signaling pathways [20]. ox-LDL markedly induced the increase of cells JNK mRNA and protein expression, which regulates intracellular redox state and causes apoptosis of the vascular endothelial cell [21].

Endogenous opioid peptides are neurotransmitter and neuromodulator, which widely exist in human and animal body and have many physiological and pharmacological functions. With the further research on opioid system, not only can the endogenous opioid peptides be used to inhibit central pain, but its role in regulating cardiovascular activity is much more increasingly concerned. It has been proved that endogenous opioid peptides system plays a very important role in regulating the cardiovascular of different kinds of biological species. EM1 and EM2 are *μ*-opioid receptor selective ligands which were discovered from ox brain by Zadina et al. in 1997 and its performance is similar to morphine [12]. EM1 and EM2 can reduce system arterial pressure (SAP) of the cat [22], rabbit [23], rats [24], and mice [25]. Akil [26] considered that the decompression function of EM1 and EM2 is induced mainly by releasing NO from animal endothelial cells. Champion et al. [27] are supportive of this point. From the experiment of perfusing rat hind limb, they found that EM1 and EM2 can reduce hind leg perfusion pressure dose-dependently, which was caused by releasing NO from vascular endothelial cell. The effect can be weakened by NO synthase inhibitors (L-NNA).

This study found that EM1 and EM2 prevented the decrease in the cell viability in the presence of ox-LDL and increased secretion of NO and NOS, which is consistent with the expression of eNOS mRNA. Meanwhile, EM1 and EM2 decreased the mRNA expression and secretion of ET-1 and JNK. And the effect of EM1 and EM2 on vascular endothelial cells could be enhanced by the JNK inhibitor SP600125. The research findings suggest that EM1 and EM2 can correct the endothelial cell dysfunction induced by ox-LDL and the protective effect of it may be achieved by affecting the JNK pathway.

## Supplementary Material

Data for graphing the Figures.

## Figures and Tables

**Table 1:** The positive granule area, average light density, and integrated light density of p-JNK in HUVECs induced by ox-LDL increased for 48 h and prevented by EM1/EM2 by image-pro plus. Data is expressed as mean ± SD.  ^*∗*^ *P* < 0.05 versus control. ^#^ *P* < 0.05 versus ox-LDL.

Group	Positive granule area (*μ*m^2^)	Average light density (PU)	Integrated light density (PU)
Normal culture group	470 ± 27	0.28 ± 0.07	4.56 ± 0.38
Cultured in ox-LDL group	4753 ± 76^*∗*^	0.84 ± 0.05^*∗*^	31.44 ± 0.74^*∗*^
EM1 (10 nmol/L) group	3242 ± 64	0.79 ± 0.05	26.03 ± 0.67
EM1 (100 nmol/L) group	2036 ± 69^#^	0.74 ± 0.06^#^	19.87 ± 0.51^#^
EM1 (1000 nmol/L) group	1228 ± 87^#^	0.68 ± 0.06^#^	13.11 ± 0.63^#^
EM1 (10000 nmol/L) group	1143 ± 77^#^	0.56 ± 0.08^#^	11.89 ± 0.57^#^
10.0 *μ*M SP600125+EM1 (10000 nmol/L)	873 ± 68^#^	0.41 ± 0.07^#^	8.23 ± 0.41^#^
EM2 (10 nmol/L) group	3223 ± 70	0.83 ± 0.05	25.74 ± 0.54
EM2 (100 nmol/L) group	1916 ± 65^#^	0.72 ± 0.03^#^	18.94 ± 0.47^#^
EM2 (1000 nmol/L) group	1079 ± 47^#^	0.62 ± 0.06^#^	12.03 ± 0.51^#^
EM2 (10000 nmol/L) group	957 ± 59^#^	0.53 ± 0.06^#^	10.81 ± 0.43^#^
10.0 *μ*M SP600125+EM2 (10000 nmol/L)	769 ± 46^#^	0.38 ± 0.05^#^	7.56 ± 0.39^#^
