# Supplementary material for: Effect of Endomorphins on HUVECs Treated by ox-LDL and Its Related Mechanisms
Source: J Diabetes Res. 2016 Aug 4;2016:9741483. doi: 10.1155/2016/9741483 (PMC4989076; doi:10.1155/2016/9741483)
Supplement: Supplementary file 1 — Data for graphing the Figures. [file 9741483.f1.docx]

**Fig.1.** cell viability

| grouping | cell viability（%）（EM1） | | |
| --- | --- | --- | --- |
| normal | |  | 99.14±9.67 |
| Ox-LDL | |  | 53.18±7.48 |
| Ox-LDL +EM1(10nM)) | |  | 70.16±8.31 |
| Ox-LDL +EM1(100nM)) | |  | 71.7±7.46 |
| Ox-LDL +EM1(1000nM)) | |  | 76.19±7.34 |
| Ox-LDL +EM1(10000nM)) | |  | 79.79±6.44 |
| Ox-LDL +SP+EM1(10000nM)) | |  | 85.21 ±7.29 |

| grouping | cell viability（%）（EM2） | | |
| --- | --- | --- | --- |
| normal | |  | 99.14±9.67 |
| Ox-LDL | |  | 53.18±4.48 |
| Ox-LDL +EM2(10nM)) | |  | 68.83±5.56 |
| Ox-LDL +EM2(100nM)) | |  | 70.45±6.74 |
| Ox-LDL +EM2(1000nM)) | |  | 75.41±6.66 |
| Ox-LDL +EM2(10000nM)) | |  | 79.21±7.63 |
| Ox-LDL +SP+EM2(10000nM)) | |  | 80.63±7.74 |

**Fig.2** NOS activity(U/ml)

| grouping | NOS activity (EM1) | | |
| --- | --- | --- | --- |
| normal | |  | 0.79±0.08 |
| Ox-LDL | |  | 0.51±0.06 |
| Ox-LDL +EM1(10nM)) | |  | 0.60±0.05 |
| Ox-LDL +EM1(100nM)) | |  | 0.64±0.05 |
| Ox-LDL +EM1(1000nM)) | |  | 0.70±0.06 |
| Ox-LDL +EM1(10000nM)) | |  | 0.75±0.04 |
| Ox-LDL +SP+EM1(10000nM)) | |  | 0.77±0.05 |

| grouping | NOS activity (EM2) | | |
| --- | --- | --- | --- |
| normal | |  | 0.79±0.06 |
| Ox-LDL | |  | 0.51±0.05 |
| Ox-LDL +EM2(10nM)) | |  | 0.58±0.05 |
| Ox-LDL +EM2(100nM)) | |  | 0.61±0.07 |
| Ox-LDL +EM2(1000nM)) | |  | 0.65±0.07 |
| Ox-LDL +EM2(10000nM)) | |  | 0.71±0.06 |
| Ox-LDL +SP+EM2(10000nM)) | |  | 0.75±0.07 |

**Fig.3** NO content(mM)

| grouping | NO content (EM1) | | |
| --- | --- | --- | --- |
| normal | |  | 18.23±0.91 |
| Ox-LDL | |  | 6.63±0.63 |
| Ox-LDL +EM1(10nM)) | |  | 7.78±0.74 |
| Ox-LDL +EM1(100nM)) | |  | 11.74±0.75 |
| Ox-LDL +EM1(1000nM)) | |  | 13.46±0.83 |
| Ox-LDL +EM1(10000nM)) | |  | 16.09±0.94 |
| Ox-LDL +SP+EM1(10000nM)) | |  | 16.26±0.73 |

| grouping | NO content (EM2) | | |
| --- | --- | --- | --- |
| normal | |  | 18.23±0.81 |
| Ox-LDL | |  | 6.63±0.53 |
| Ox-LDL +EM2(10nM)) | |  | 7.61±0.62 |
| Ox-LDL +EM2(100nM)) | |  | 11.45±0.55 |
| Ox-LDL +EM2(1000nM)) | |  | 13.05±0.78 |
| Ox-LDL +EM2(10000nM)) | |  | 15.31±0.84 |
| Ox-LDL +SP+EM2(10000nM)) | |  | 16.09±0.58 |

**Fig.4**  ET-1 content(mM)

| grouping | ET-1 content（EM1） | | |
| --- | --- | --- | --- |
| normal | |  | 0.75±0.06 |
| Ox-LDL | |  | 1.57±0.09 |
| Ox-LDL +EM2(10nM)) | |  | 1.42±0.10 |
| Ox-LDL +EM1(100nM)) | |  | 1.14±0.08 |
| Ox-LDL +EM1(1000nM)) | |  | 0.91±0.07 |
| Ox-LDL +EM1(10000nM)) | |  | 0.69±0.07 |
| Ox-LDL +SP+EM1(10000nM)) | |  | 0.72±0.08 |

| grouping | ET-1 content（EM2） | | |
| --- | --- | --- | --- |
| normal | |  | 0.75±0.06 |
| Ox-LDL | |  | 1.57±0.09 |
| Ox-LDL +EM2(10nM)) | |  | 1.49±0.09 |
| Ox-LDL +EM2(100nM)) | |  | 1.31±0.08 |
| Ox-LDL +EM2(1000nM)) | |  | 1.04±0.07 |
| Ox-LDL +EM2(10000nM)) | |  | 0.82±0.08 |
| Ox-LDL +SP+EM2(10000nM)) | |  | 0.72±0.07 |

**Fig.5** eNOS mRNA

| grouping | eNOS mRNA(EM1) | | |
| --- | --- | --- | --- |
| normal | |  | 1.00±0.00 |
| Ox-LDL | |  | 0.28±0.02 |
| Ox-LDL +EM1(10nM)) | |  | 0.35±0.03 |
| Ox-LDL +EM1(100nM)) | |  | 0.49±0.03 |
| Ox-LDL +EM1(1000nM)) | |  | 0.61±0.05 |
| Ox-LDL +EM1(10000nM)) | |  | 0.71±0.04 |
| Ox-LDL +SP+EM1(10000nM)) | |  | 0.89±0.03 |

| grouping | eNOS mRNA(EM2) | | |
| --- | --- | --- | --- |
| normal | |  | 1.00±0.00 |
| Ox-LDL | |  | 0.28±0.02 |
| Ox-LDL +EM2(10nM)) | |  | 0.32±0.02 |
| Ox-LDL +EM2(100nM)) | |  | 0.43±0.04 |
| Ox-LDL +EM2(1000nM)) | |  | 0.56±0.04 |
| Ox-LDL +EM2(10000nM)) | |  | 0.68±0.07 |
| Ox-LDL +SP+EM2(10000nM)) | |  | 0.85±0.05 |

**Fig.6** ET-1mRNA

| grouping | ET-1mRNA（EM1） | | |
| --- | --- | --- | --- |
| normal | |  | 1.00±0.00 |
| Ox-LDL | |  | 2.78±0.12 |
| Ox-LDL +EM1(10nM)) | |  | 2.49±0.16 |
| Ox-LDL +EM1(100nM)) | |  | 2.19±0.11 |
| Ox-LDL +EM1(1000nM)) | |  | 1.94±0.09 |
| Ox-LDL +EM1(10000nM)) | |  | 1.60±0.13 |
| Ox-LDL +SP+EM1(10000nM)) | |  | 1.48±0.14 |

| grouping | ET-1mRNA（EM2） | | |
| --- | --- | --- | --- |
| normal | |  | 1.00±0.00 |
| Ox-LDL | |  | 2.78±0.14 |
| Ox-LDL +EM2(10nM)) | |  | 2.53±0.17 |
| Ox-LDL +EM2(100nM)) | |  | 2.27±0.20 |
| Ox-LDL +EM2(1000nM)) | |  | 2.02±0.09 |
| Ox-LDL +EM2(10000nM)) | |  | 1.68±0.12 |
| Ox-LDL +SP+EM2(10000nM)) | |  | 1.52±0.11 |

**Fig.7** JNK mRNA

| grouping | JNK mRNA(EM1) | | |
| --- | --- | --- | --- |
| normal | |  | 1.00±0.00 |
| Ox-LDL | |  | 2.91±0.22 |
| Ox-LDL +EM1(10nM)) | |  | 2.76±0.19 |
| Ox-LDL +EM1(100nM)) | |  | 2.25±0.17 |
| Ox-LDL +EM1(1000nM)) | |  | 1.84±0.14 |
| Ox-LDL +EM1(10000nM)) | |  | 1.38±0.15 |
| Ox-LDL +SP+EM1(10000nM)) | |  | 1.24±0.18 |

| grouping | JNK mRNA(EM2) | | |
| --- | --- | --- | --- |
| normal | |  | 1.00±0.00 |
| Ox-LDL | |  | 2.91±0.21 |
| Ox-LDL +EM2(10nM)) | |  | 2.80±0.19 |
| Ox-LDL +EM2(100nM)) | |  | 2.37±0.16 |
| Ox-LDL +EM2(1000nM)) | |  | 1.96±0.12 |
| Ox-LDL +EM2(10000nM)) | |  | 1.49±0.14 |
| Ox-LDL +SP+EM2(10000nM)) | |  | 1.30±0.15 |
